# Supplementary material for: Hotspot motion caused the Hawaiian-Emperor Bend and LLSVPs are not fixed
Source: Nat Commun. 2019 Jul 29;10:3370. doi: 10.1038/s41467-019-11314-6 (PMC6662702; doi:10.1038/s41467-019-11314-6)
Supplement: Supplementary file 1 — Supplementary Information [file 41467_2019_11314_MOESM1_ESM.pdf]

Supplementary Information for

Hotspot motion caused the Hawaiian-Emperor Bend and LLSVPs are not fixed

Richard K. Bono, John A. Tarduno, Hans-Peter Bunge

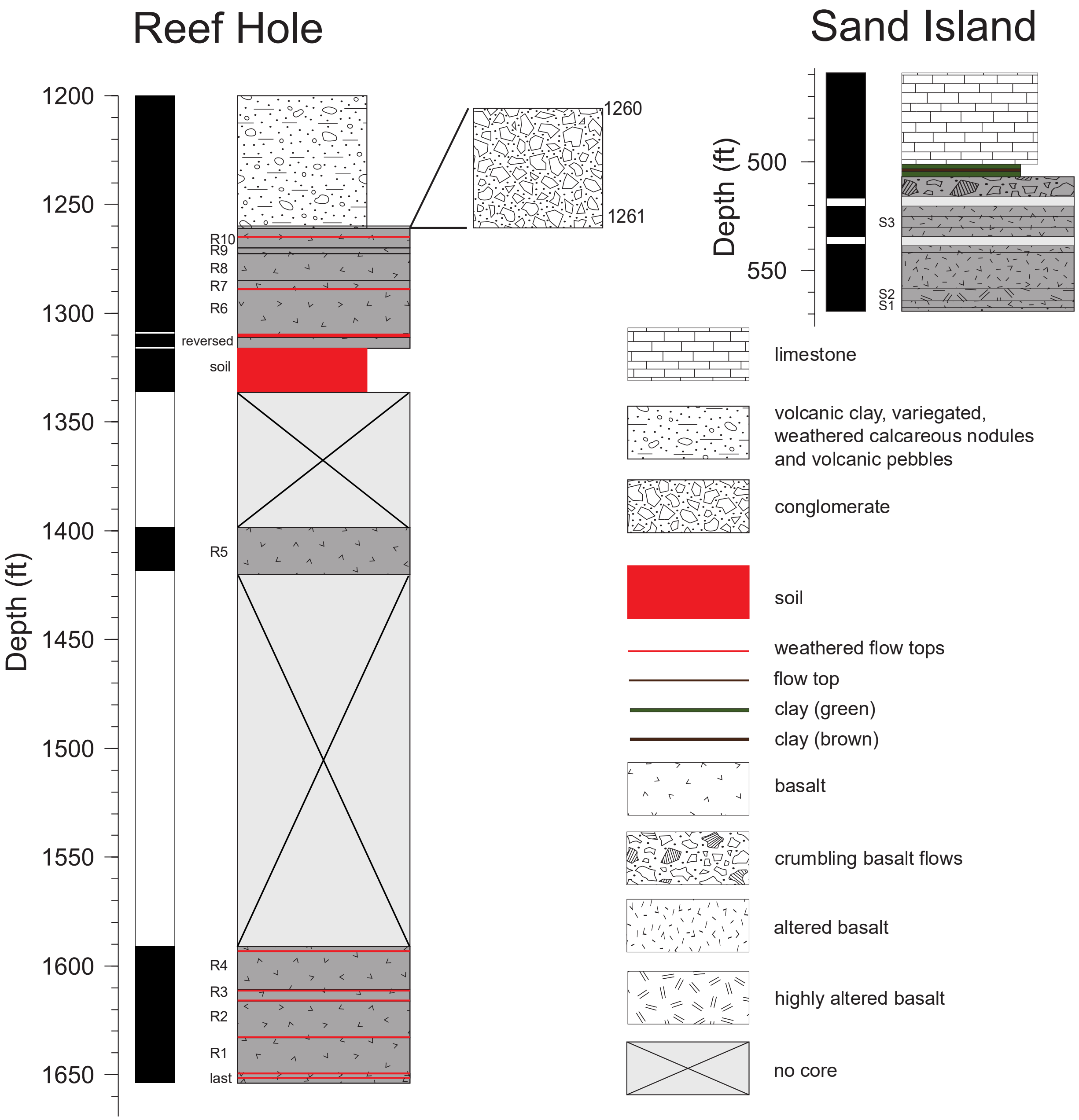

Supplementary Figure 1: Stratigraphy of the basement sections of the Midway Atoll. Lithology of volcanic units recovered from Reef and Sand Island drill cores[1−2]. Depth scale reported in feet, preserving the unit of record. Black bar between depth scale and stratigraphy shows depths where core was successfully recovered. Regions marked in the stratigraphy with a black cross over a grey field are depths in which a rock bit was used instead of a coring bit.

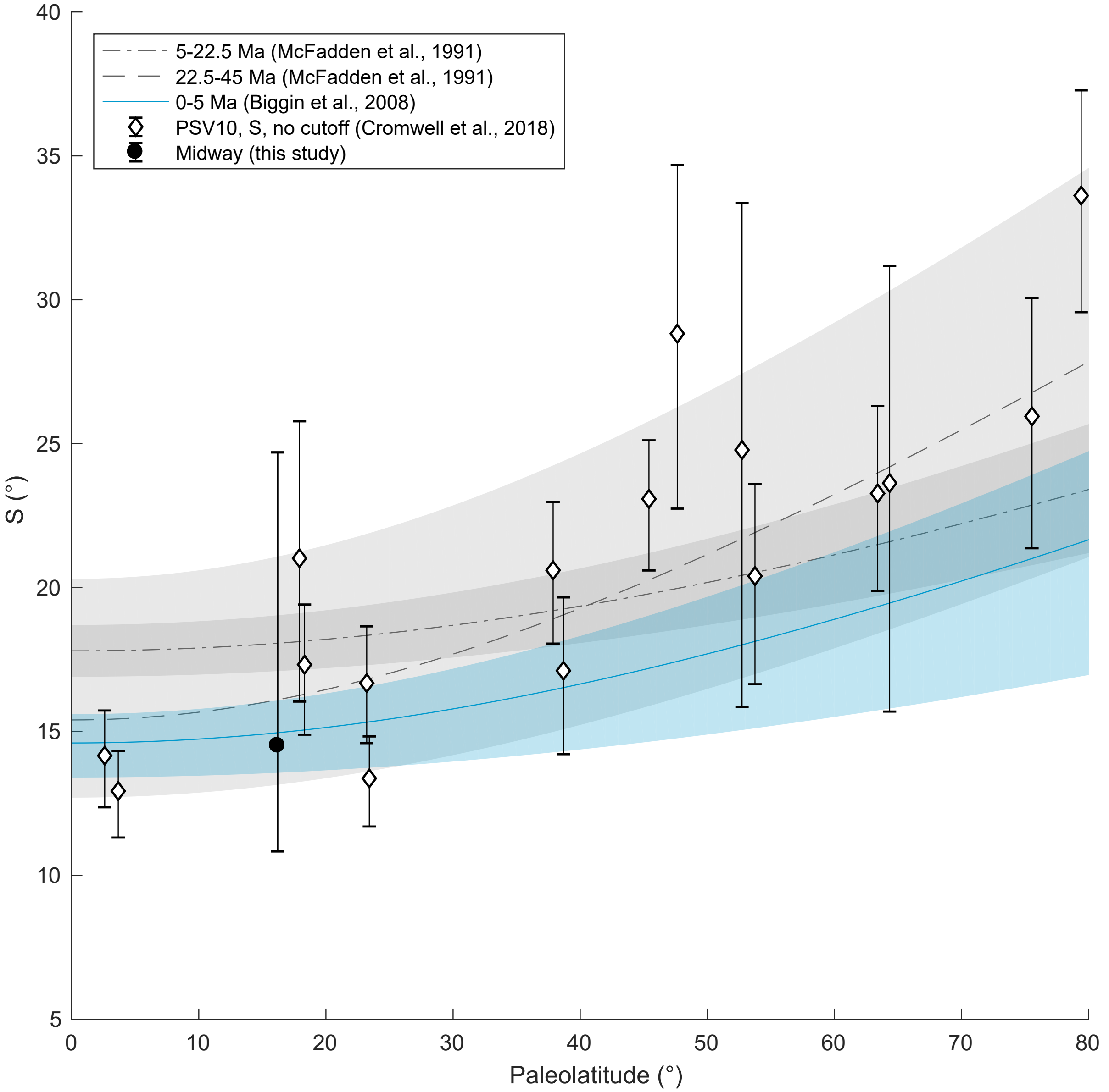

Supplementary Figure 2: Virtual geomagnetic dispersion (S) plotted against paleolatitude inferred from paleomagnetic observations for this study and corresponding time periods. Filled black circle represents S for 28 Ma from this study (Methods), with 95% confidence intervals from bootstrap analysis[3]. Open diamonds show S (calculated without a cut-off angle) for the past 10 Ma with 95% confidence intervals from a bootstrap analysis[4]. Blue line and shaded region show Model G fit[5] (with 95% confidence) for the past 5 Ma[6]. Grey dot-dashed line with shaded region of Model G fit and 95% confidence region for 5 to 22.5 Ma[5]; grey dashed line shows Model G fit for 22.5 to 45 Ma[5]. Model G fits of ref. (5) have not been corrected for use of a constant within-site dispersion, assumed to introduce only a minor component of variation.

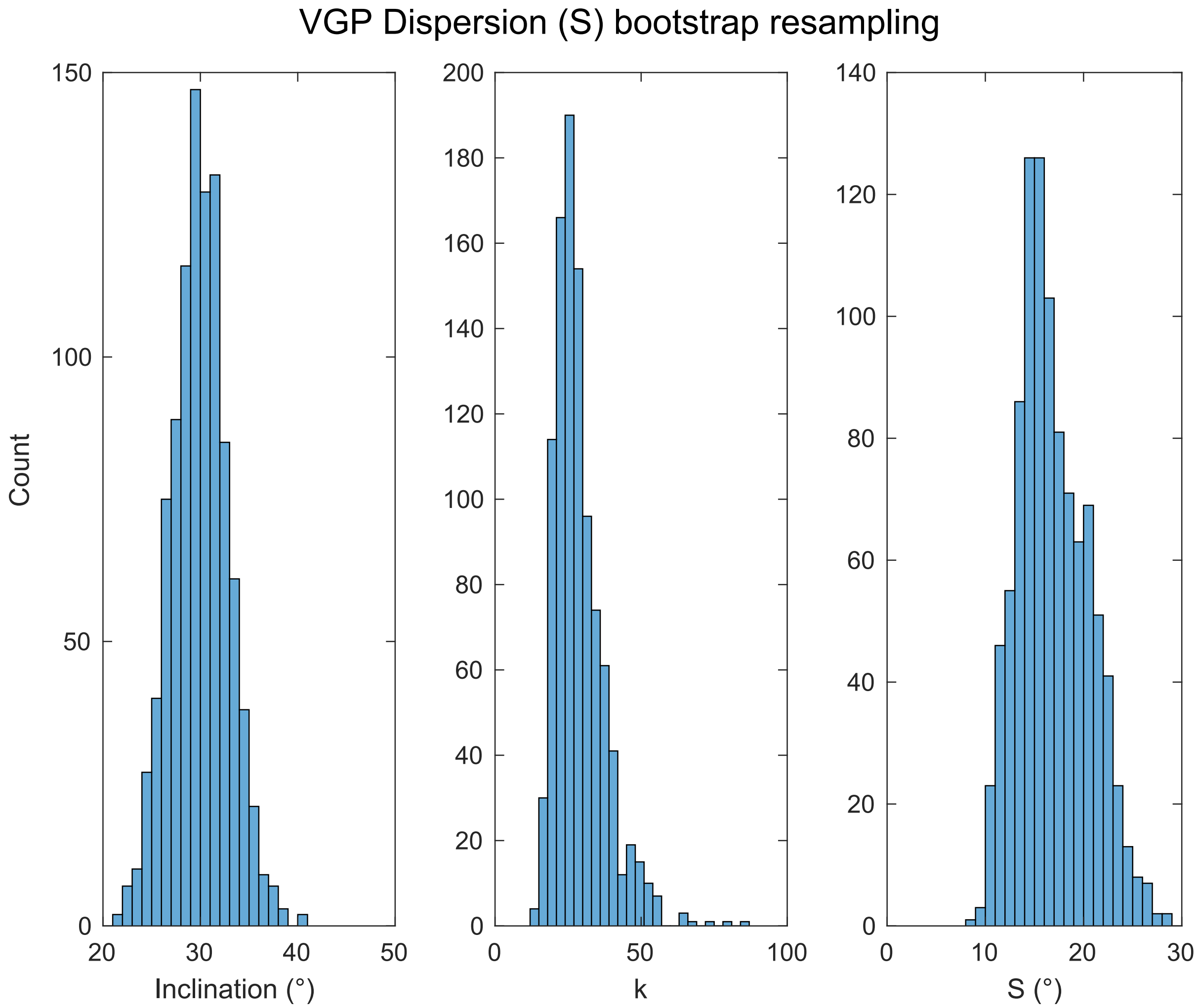

Supplementary Figure 3: Distribution of inputs and calculated VGP dispersion (S) from bootstrap resampling. Left panel shows the distribution of mean inclinations (in degrees) for all draws; center panel shows the distribution of precision parameter k; right panel shows the distribution of VGP dispersion (S, degrees).

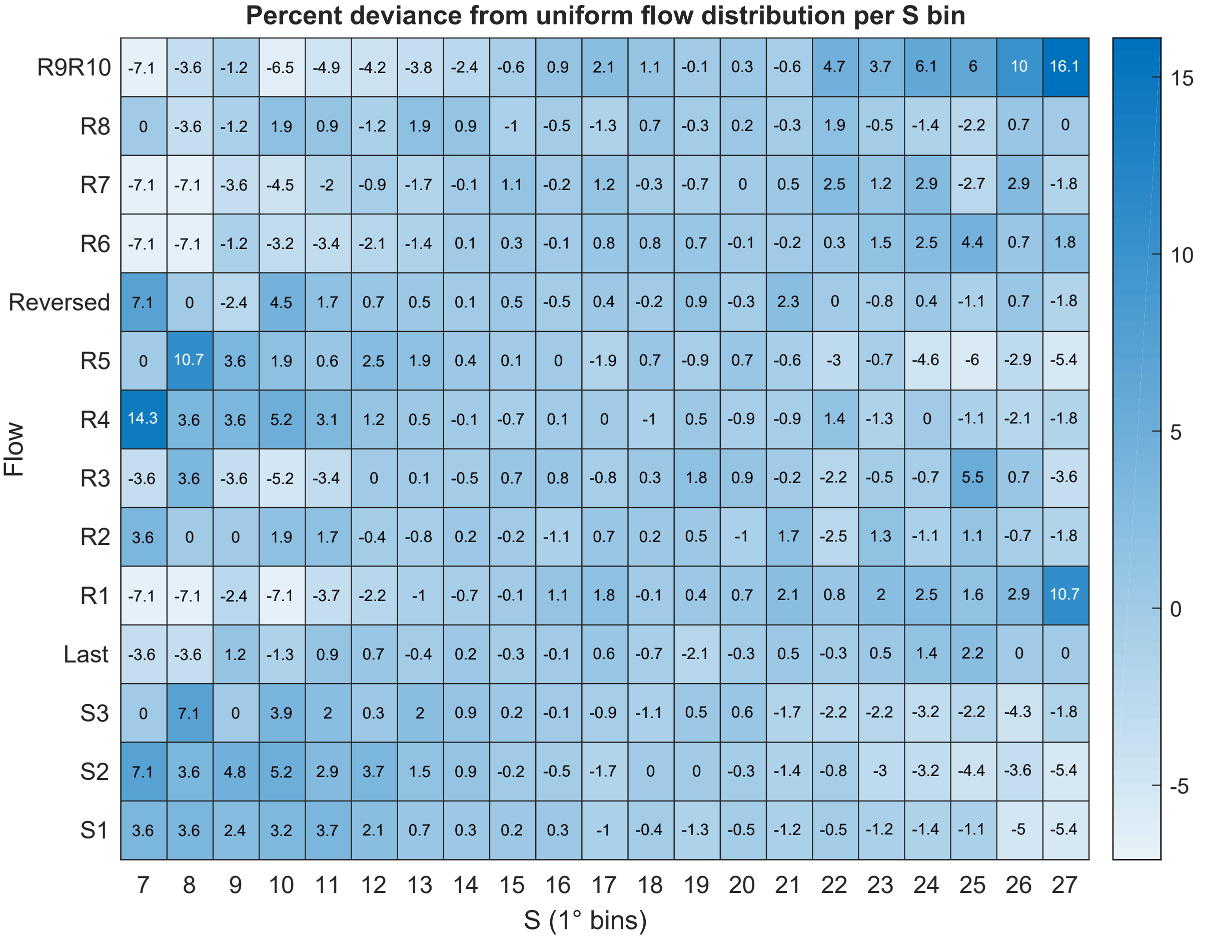

Supplementary Figure 4: Heat map of relative percent deviance from a uniform flow distribution per VGP dispersion (S) bin following bootstrap resampling. VGP dispersion (S) is divided into 1 degree bins. Intensity of color and numeric value in each cell corresponds with the percent deviation away from a uniform distribution of flows contributing to a given S bin. High values may point to a limited number of lavas.

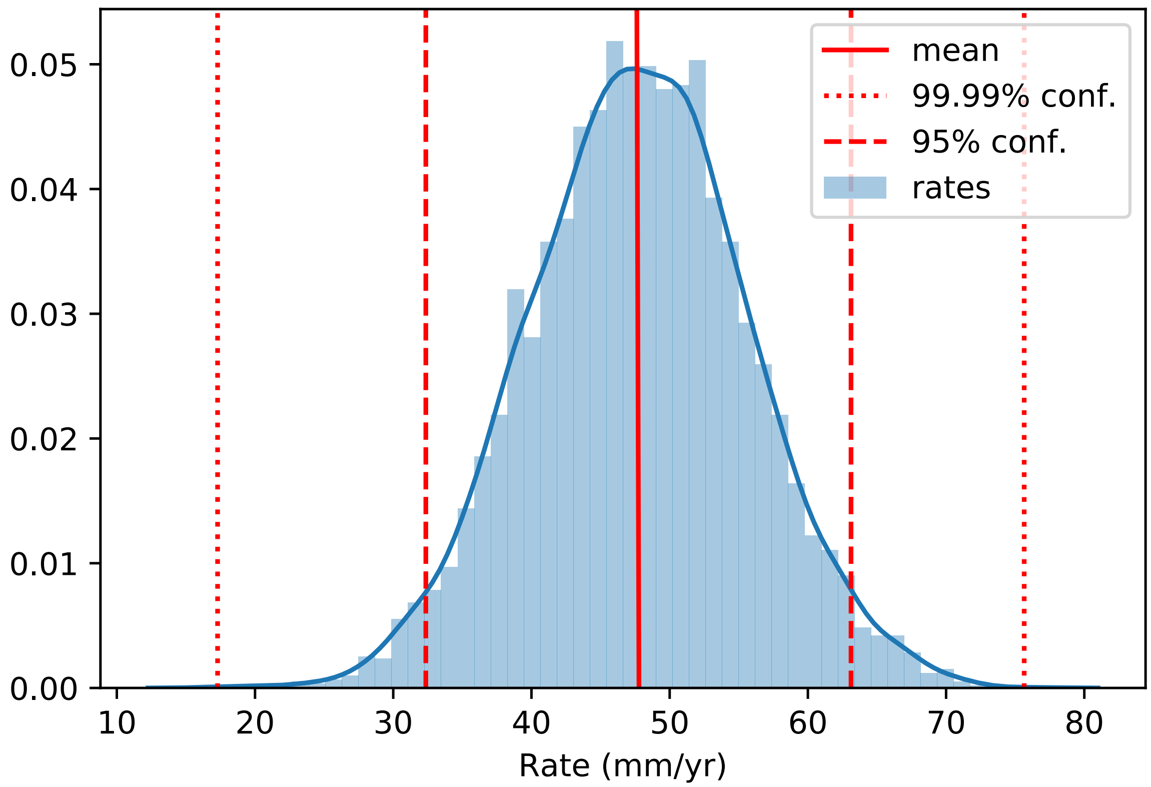

Supplementary Figure 5: Markov Chain Monte Carlo (MCMC) analysis of rate of hotspot motion. Distribution of posterior estimates of the rate of motion of the Hawaiian hotspot based on paleomagnetic paleolatitude values; blue line shows kernel density estimate of distribution. Mean rate (solid red line), 95% confidence (dashed red line) and 99.99% confidence (dotted red line) intervals shown.

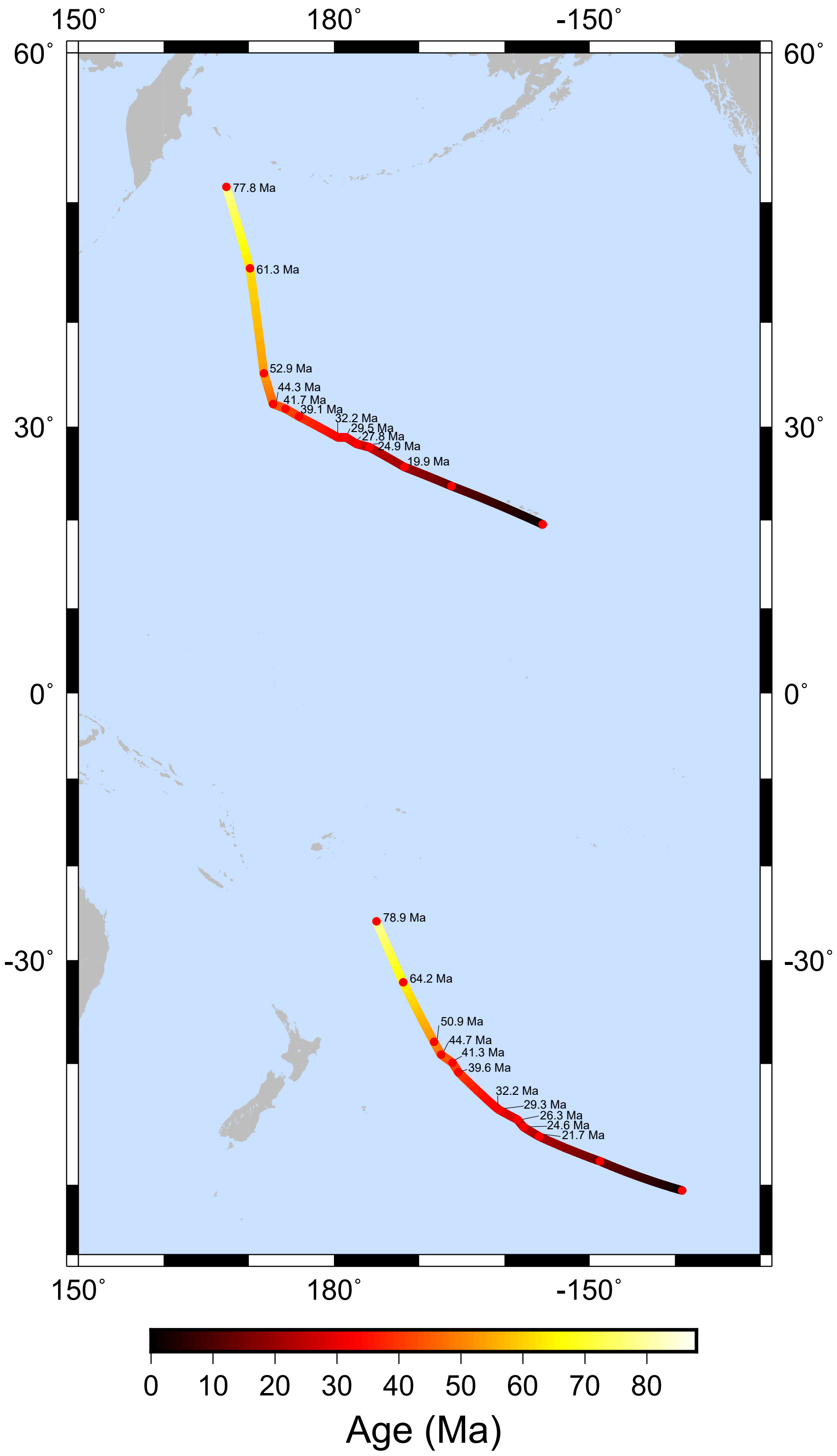

Supplementary Figure 6: Select Louisville and Hawaii-Emperor track seamounts. Seamounts used to calculate changes in distance between the Hawaiian and Louisville hotspots. Although it does not factor in our distance calculations, we note that the youngest Louisville location shown is 1.1 Ma from Koppers et al.[7].

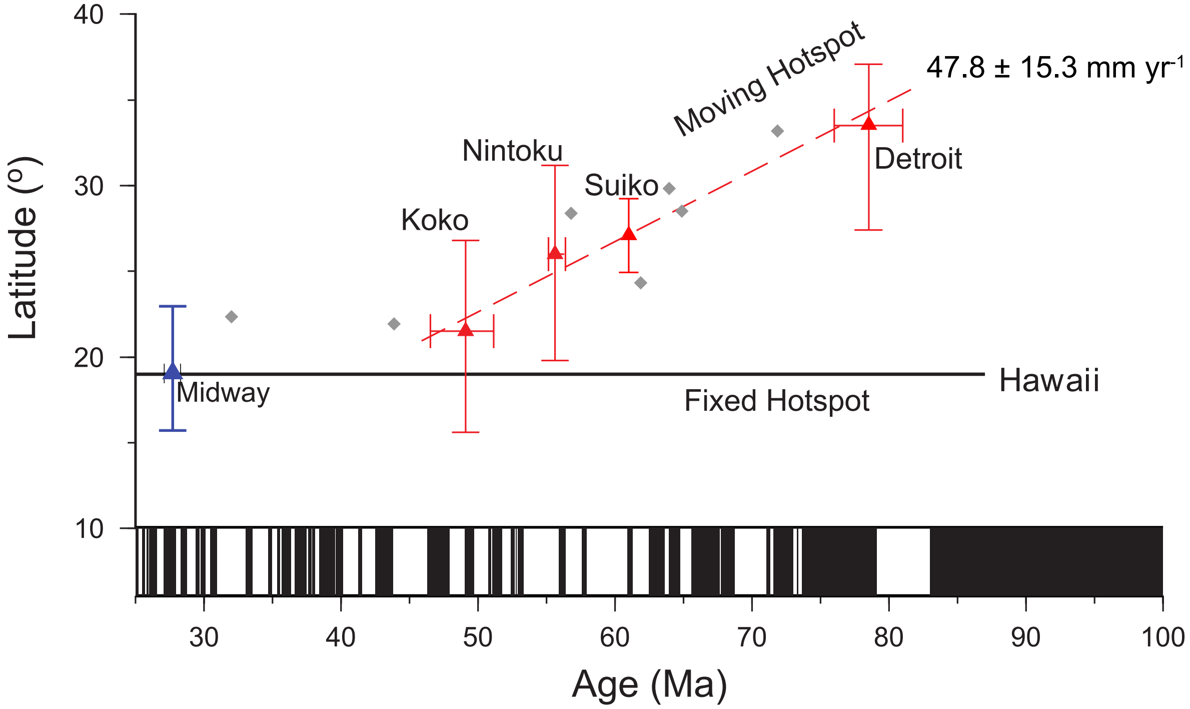

Supplementary Figure 7: Skewness model poles predictions versus versus paleomagnetic data. Red: test of hotspot fixity based on time-averaged data from paleomagnetic analyses of samples recovered from scientific drilling of seamounts[8] together with paleolatitude value from Midway Atoll (blue triangle) presented here. Grey: marine magnetic anomaly skewness model pole predictions[9–14]. As noted in the main text, the closest skewness-based modeled paleolatitude to the age of Midway comes from a 32 Ma values (marine magnetic anomaly chron 12r). Horner-Johnson and Gordon[13] reported a model pole (83.5 ^{o}N, 44.6 ^{o}E) which yields an expected paleolatitude of 24.2^o, with an extraordinarily small (approximately $\pm 1.3^o$) model uncertainty. We note that this uncertainty cannot be reproduced from the relevant figure in ref. (13) which must represent a plotting error. Subsequently, Zheng et al.[9] revised the model pole position (82.7 ^{o}N, 26.6 ^{o}E), incorporating a 5% non-dipole (specifically quadrupole) term. This new model pole yields a paleolatitude of 22.3 N with model uncertainties of approximately $^{+1.4^o}_{-1.5^o}$ and a smaller discrepancy with the latitude of Hawaii.

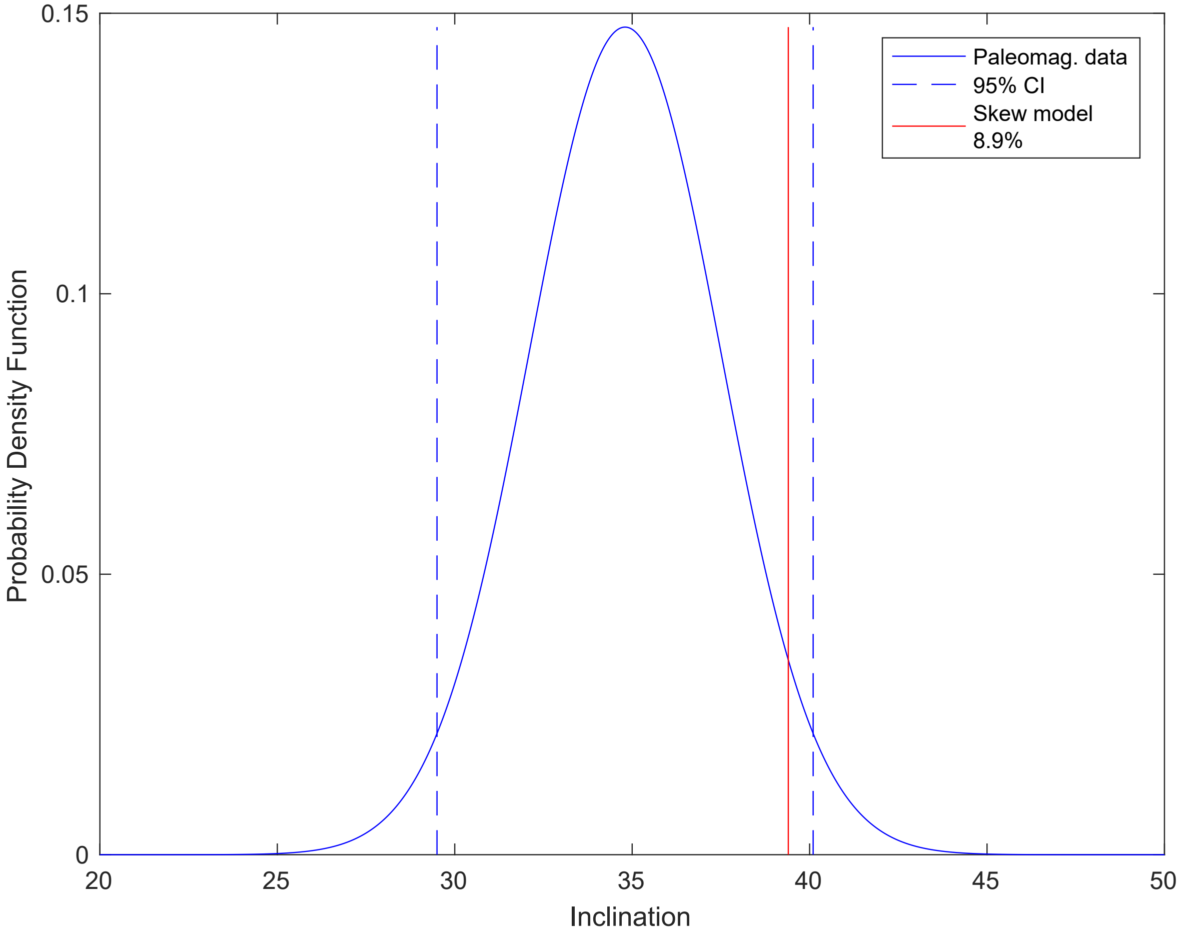

Supplementary Figure 8: Bayesian analysis of predicted inclinations from skewness models. Probability of observing predicted inclination from skewness models[9] for 32 Ma given observed distribution of paleomagnetic inclination data from Midway Atoll presented here, assuming inclinations are normally distributed. Paleomagnetic mean inclination (solid line) and 95% confidence intervals (dashed line) plotted in blue. Skewness model inclination as red line, with probability of observing skewness model inclination in legend.

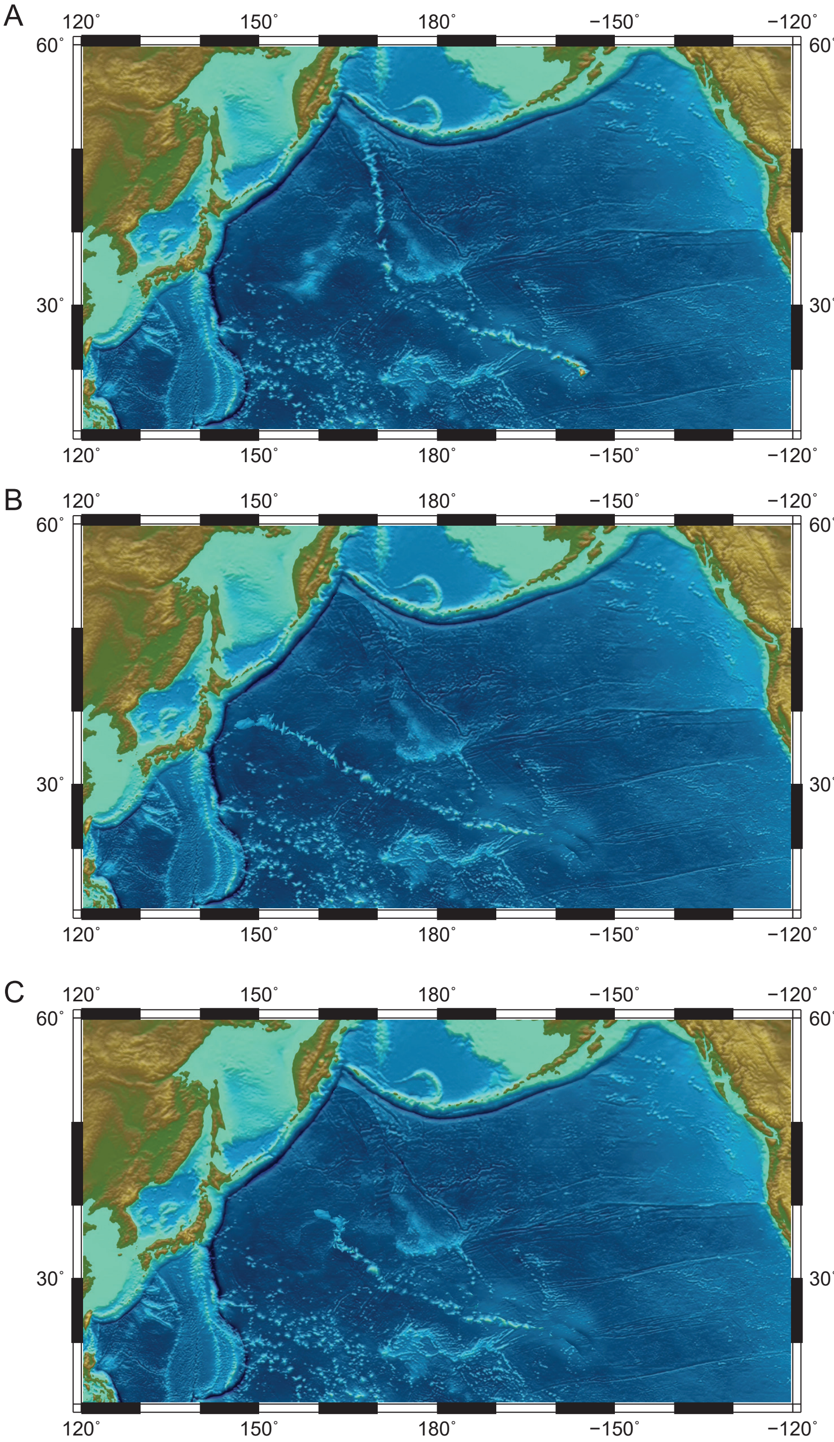

Supplementary Figure 9: Predicted morphologies of Hawaiian-Emperor track had the Hawaiian plume been fixed in the mantle[15−16]. (A) Present-day bathymetry of North Pacific Basin. (B) Synthetic bathymetry for Hawaiian-Emperor seamounts as predicted by East-West Antarctica[17] plate circuit model; (C) predictions based on Australia-Lord Howe Rise plate circuit[18] modified with revised Antarctic-Australia spreading history[19−20].

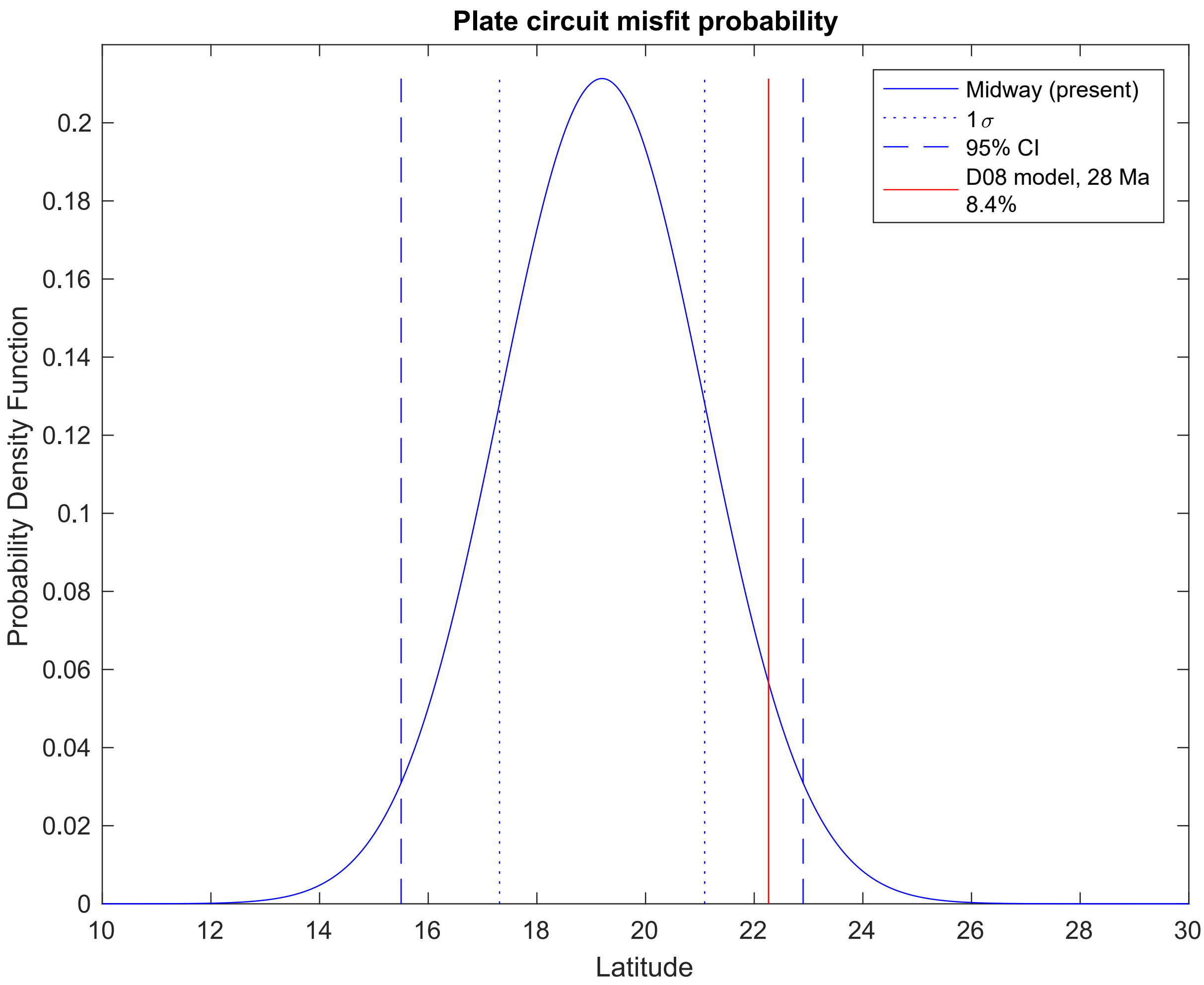

Supplementary Figure 10: Bayesian analysis of predicted paleolatitude for Midway Atoll based on plate circuits compared to observed paleomagnetic paleolatitude. A normal distribution of paleolatitudes with the same mean value as observed in paleomagnetic data for Midway Atoll presented here, with variance defined by upper 95% confidence interval from the paleomagnetic data shown in blue. Red: predicted paleolatitude for Midway Atoll based on plate circuit analyses[21–22]. Probability of observing paleolatitude predicted by plate circuit given distribution of paleomagnetic paleolatitudes observed in legend.

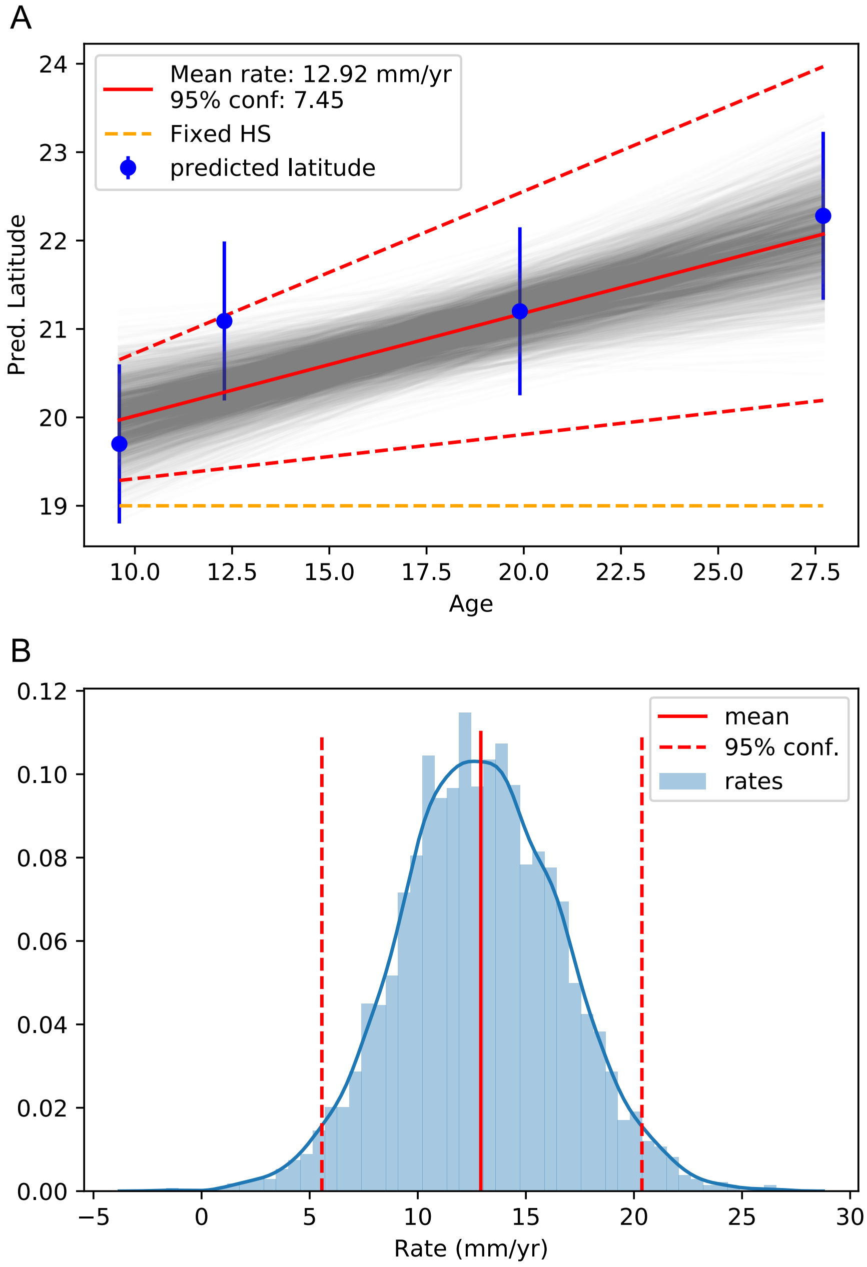

Supplementary Figure 11: Bayesian Markov Chain Monte Carlo estimation of rate of LLSVP wander. (A) Predicted latitude for Hawaiian seamounts using a fixed Indo-Atlantic hotspot reference frame and plate circuits. Blue circles: predicted latitude using plate circuits[21–22] with 95% confidence intervals. Red line: mean estimated linear rate to satisfy the observed predicted latitudes, dashed lines: 95% confidence interval. Orange dashed line: fixed paleolatitude for Hawaiian hotspot. Grey lines: 2500 individual model realizations. (B) Histogram of posterior probability distribution of MCMC modeled rates (in mm/yr) which satisfy the plate circuit paleolatitude predictions, with mean and 95% confidence ranges in red lines (solid and dashed, respectively). Blue line: kernel density estimate of the posterior probability.

Supplementary Table 1: Summary table of Reef and Sand Island hole inclination-only flow averages

Flow	Inc. (°)	N	k	α_{95}(°)
R9R10[a]	9.5	6	198	5.3
R8	39.3	6	125	6.6
R7	43.5	6	197	5.3
R6	44.6	5	229	5.9
Rev	-19.3	6	132	6.5
Soil	39.9	13	41	6.4
R5	30.3	6	357	3.9
R4	23.7	7	67	7.9
R3	16.8	5	663	3.5
R2	20.2	7	250	4.1
R1	45.8	6	101	7.4
Last	40.7	5	182	6.7
S3	29.7	8	145	4.8
S2	30.9	6	93	7.7
S1	26.7	6	107	7.2

[a] Flows R9 and R10 have been grouped together into Flow R9R10 for paleomagnetic analysis due to similar inclination values suggesting insufficient time passed between flows to average the geomagnetic field.

Supplementary Table 2: Paleomagnetic results from Reef and Sand Island Holes

Flow	Specimen	Steps Fit (+Origin)[a]	n[b]	Dec. (°)[c]	Inc. (°)	MAD (°)[d]
R10	MR335-29	550–625+O	5	60.5	8.4	2.6
R10	MR335-47	525–625+O	6	52.8	9.1	2.4
R10	MR336-24	525–625+O	6	244.3	8.5	1.9
R9	MR337-22	525–625+O	6	330.7	9.2	3.2
R9	MR337-26	500–625+O	7	199.9	4.9	4.0
R9	MR337-48	500–625+O	7	299.5	17.2	0.4
R8	MR338-33	500–625+O	7	32.9	39.4	3.4
R8	MR338-46	550–625+O	5	141.9	42.3	1.2
R8	MR339-14	450–625+O	9	76.1	40.0	2.3
R8	MR339-31	525–625+O	6	250.6	45.7	1.0
R8	MR339-8	550–625+O	5	232.5	30.4	0.8
R8	MR339-9	425–625+O	12	42.3	38.0	1.9
R7	MR340-47	450–625+O	9	316.8	39.7	2.5
R7	MR341-6	525–625+O	6	235.1	45.4	1.5
R7	MR341-11	550–625+O	5	136.1	41.6	2.1
R7	MR341-16	550–625+O	5	281.0	38.7	1.4
R7	MR341-40	550–625+O	5	220.5	47.2	2.4
R7	MR341-44	500–625+O	7	299.8	48.5	1.4
R6	MR342-37	375–625+O	12	10.2	48.9	8.9
R6	MR342-38	p250–625+O	17	161.2	48.3	5.3
R6	MR343-38	400–625+O	11	7.8	43.4	3.3
R6	MR343-39	525–625+O	6	13.8	40.5	2.4
R6	MR344-18	500–625+O	7	74.7	42.0	6.9
Contact[e]	MR345-3	400–675+O	13	248	22.9	8.8
Contact	MR345-4	500–625+O	7	44.3	-59.8	5.3
Reversed	MR345-11	550–675+O	7	324.7	-16.7	2.4
Reversed	MR345-12	425–625+O	10	317.9	-15.6	4.5
Reversed	MR345-15	550–675+O	7	214.1	-24.0	3.3
Reversed	MR345-23	425–675+O	12	92.0	-18.1	3.5
Reversed	MR345-45	525–625+O	6	56.6	-14.5	3.8
Reversed	MR345-52	525–675+O	8	80.0	-26.9	3.2
Contact	MR346-11	425–600+O	9	277.9	-41.5	12.0
Contact	MR346-21	525–625+O	6	272.1	29.8	5.0
Soil	MR346-37	550–675+O	7	136.2	37.6	2.8
Soil	MR346-56	550–675+O	7	123.3	39.4	2.6
Soil	MR347-8	550–690+O	8	151.6	40.6	4.1
Soil	MR347-14	525–690+O	9	245.9	35.7	2.5
Soil	MR347-20	550–690+O	8	299.5	36.7	2.3
Soil	MR347-47	500–600+O	6	269.0	28.4	4.6
Soil	MR347-55	525–690+O	9	212.1	39.1	1.7
Soil	MR348-12	500–690+O	10	305.0	24.6	1.1
Soil	MR348-19	550–690+O	8	350.0	58.9	1.8
Soil	MR348-24	550–690+O	8	44.7	45.1	1.3
Soil	MR348-30	525–690+O	9	304.8	53.1	2.1
Soil	MR348-37	525–650	6	191.2	40.7	1.3
Soil	MR348-41	550–650+O	6	208.8	39.4	2.5

Supplementary Table 2 continued: Paleomagnetic results from Reef and Sand Island Holes

Flow	Specimen	Steps Fit (+Origin)[a]	n[b]	Dec. (°)[c]	Inc. (°)	MAD (°)[d]
R5	MR351-12	400–625+O	11	8.5	34.3	3.5
R5	MR351-13	200–625	18	4.1	32.3	2.9
R5	MR352-10	525–625+O	6	136.7	29.1	2.2
R5	MR352-27	500–625+O	7	335.4	31.7	1.8
R5	MR354-6	325–625+O	14	355.4	26.2	4.6
R5	MR354-7	500–625+O	7	354.8	27.9	2.1
R4	MR356-25	525–625+O	6	120.6	19.4	4.4
R4	MR356-26	500–625+O	7	114.2	17.7	3.1
R4	MR356-30	550–625+O	5	301.7	13.8	12.3
R4	MR357-20	550–625+O	5	199.3	29.2	1.5
R4	MR358-30	550–625+O	5	1.9	23.6	1.6
R4	MR359-6	525–625+O	6	291.9	31.0	3.3
R4	MR359-30	550–625+O	5	49.3	31.2	1.0
Contact[e]	MR359-35	550–625+O	5	31.0	25.8	1.5
R3	MR359-46	500–625+O	7	297.1	19.0	1.9
R3	MR359-47	500–625	6	106.5	16.4	2.0
R3	MR359-48	550–625+O	5	104.1	16.9	1.8
R3	MR359-57	500–625+O	7	8.4	18.4	1.0
R3	MR359-58	375–625	11	9.0	13.3	3.2
R2	MR361-22	500–625+O	7	290.3	20.0	1.1
R2	MR362-6	525–625+O	6	96.2	16.6	5.1
R2	MR362-12	425–625	9	168.9	22.1	15.2
R2	MR362-13	500–625+O	7	248.2	24.9	6.0
R2	MR362-18	550–625+O	5	245.4	22.0	2.1
R2	MR363-33	450–625+O	9	36.0	14.3	6.5
R2	MR363-34	500–625+O	7	53.5	21.6	6.6
R1	MR365-24	525–625+O	6	107.5	47.8	3.4
R1	MR365-49	475–625+O	8	332.3	40.2	1.9
R1	MR365-50	525–625+O	6	335.1	41.0	3.2
R1	MR365-55	500–625+O	7	51.1	41.4	1.9
R1	MR366-28	525–625+O	6	177.2	53.7	1.9
R1	MR367-21	450–625+O	9	331.7	50.5	3.4
Contact	MR368-15	550–625+O	5	126.0	61.6	1.2
Last	MR368-21	500–625+O	7	231.0	47.7	1.1
Last	MR368-25a	525–625	5	16.3	37.1	3.3
Last	MR368-25b	525–600	4	271.2	41.1	7.6
Last	MR368-27	400–625+O	11	13.3	37.6	2.4
Last	MR368-28	400–625+O	11	13.2	40.0	4

Supplementary Table 2 continued: Paleomagnetic results from Reef and Sand Island Holes

Flow	Specimen	Steps Fit (+Origin)[a]	n[b]	Dec. (°)[c]	Inc. (°)	MAD (°)[d]
S3	S87-31	525–625+O	6	36.3	30.9	1.1
S3	S87-32	525–625+O	6	36.5	27.6	0.9
S3	S87-33	550–625+O	5	37.5	22.3	2.2
S3	s87-58	550–625	4	205.1	26.1	3.8
S3	s88-12	525–625+O	6	83.3	27.2	2.7
S3	S88-31	550–625+O	5	127.4	36.2	2.4
S3	S88-32	525–625+O	6	304.8	35.4	2.7
S3	S88-33	550–625+O	5	128.8	31.7	3.2
S2	S90-11	550–625	4	280.5	35.5	3.0
S2	S90-12	500–625+O	7	152.4	26.0	3.3
S2	S90-23	525–625+O	6	231.6	35.8	1.9
S2	S90-40	450–625+O	9	277.1	37.0	3.3
S2	S90-41	550–625+O	5	150.9	28.0	3.8
S2	S90-51	525–625+O	6	144.7	23.0	3.4
Contact[e]	S91-23	550–625+O	5	63.3	8.5	1.2
S1	S91-24	350–625+O	13	338.1	30.1	2.0
S1	S91-25	550–625+O	5	334.7	30.0	2.8
S1	S91-26	525–625+O	6	332.7	16.7	1.5
S1	S91-33	425–625+O	10	235.4	30.9	7.4
S1	S91-34	525–625	5	249.1	23.7	4.6
S1	S91-35	525–625+O	6	52.5	28.9	1.1

[a] Unblocking temperature range of characteristic remanence fit using principal component analysis; "+O" denotes that origin is included in fit direction. [b] Number of temperature steps included in principal component fit. [c] Drill cores are azimuthally unoriented so declination cannot be used for interpretation. [d] MAD: maximum angular deviation. [e] Specimens marked as "Contact" are excluded from inclination analyses. These zones are at boundaries between flows, or between flows and soils/weathered flow tops, where multiple magnetizations are interpreted to be present.

Supplementary Table 3: Inclination-only means

	Inc. (°)	N	k	α_{95} (°)	Paleo-latitude (°)
Basalts	30.1	14[a]	25.0	7.8	$16.2\ ^{+5.1}_{-4.6}$
Basalts and soils	34.8	27	25.8	5.3	$19.2\ ^{+3.7}_{-3.4}$

[a] Number of cooling units in mean.

Supplementary Table 4: Age-location pairs for great circle distance analysis

Louisville Seamounts					*Hawaiian-Emperor Seamounts*				
Lat. (°N)	Lon. (°E)	Age (Ma)[a]	2σ	Name	Lat. (°N)	Lon. (°E)	Age (Ma)[a]	2σ	Name
-44.84	-158.47	26.3	0.3	158.5 °W	28.23	-177.37	27.8	0.9	Midway
-44.00	-160.66	29.3	0.3	160.7 °W	28.91	-178.61	29.5	0.7	Seamount #63
-43.55	-161.41	32.2	0.3	161.5 °W	28.90	-179.56	32.2	0.9	Helsley
-40.74	-165.40	39.6	0.8	165.4 °W	31.02	175.90	39.1	0.2	Colohan
-39.89	-166.10	41.3	0.3	166.1 °W	31.81	174.30	41.7	0.7	Abbot
-39.15	-167.43	44.7	0.4	167.4 °W	32.28	172.85	44.3	0.7	North Kammu
-37.97	-168.27	50.9	0.5	168.3 °W	35.28	171.77	52.9	0.8	Koko N. (shield)
-32.24	-171.89	64.2	0.5	Burton Guyot	44.73	170.13	61.3	0.3	Suiko (shield)
-25.97	-175.02	78.9	1.3	LOU-4	51.14	167.36	77.8	1.4	Detroit

[a] All Ar/Ar ages recalibrated following ref. 23.

Supplementary References

1. Ladd, H.S., Tracey, J.I. & Gross, M.G. Drilling on Midway Atoll, Hawaii. *Science* **156**, 1088-1094 (1967).
2. Macdonald, G.A. Petrology of the basalt cores from Midway Atoll. *U.S. Geol. Surv. Prof. Pap.* **680-B**, B1-B10 (1969).
3. Efron, B., & Tibshirani, R. An introduction to the bootstrap. Chapman & Hall, New York (1993).
4. Cromwell, G., Johnson, C.L., Tauxe, L., Constable, C.G., & Jarboe, N.A. PSV10: A global data set for 0-10 Ma time-averaged field and paleosecular variation studies. *Geochem. Geophy. Geosyst.*, **19**, 1533-1558 (2018).
5. McFadden, P. L., Merrill, R.T., McElhinny, M.W. & Lee, S. Reversals of the Earth's magnetic field and temporal variations of the dynamo families. *J. Geophys. Res.* **96**, 3923-3933 (1991).
6. Biggin, A.J., van Hinsbergen, D.J.J., Langereis, C.G., Straathof, G.B., & Deenen, M.H.L. Geomagnetic secular variation in the Cretaceous Normal Superchron and in the Jurassic. *Phys. Earth Planet. Inter.* **169** 3-19 (2008).
7. Koppers, A.A.P., *et al.* New ^{40}Ar/^{39}Ar age progression for the Louisville hot spot trail and implications for inter-hot spot motion. *Geochem. Geophys. Geosyst.* **12**, Q0AM02 (2011).
8. Tarduno, J.A., *et al.* The Emperor seamounts: Southward motion of the Hawaiian hotspot plume in Earth's mantle. *Science* **301**, 1064-1069 (2003).
9. Zheng, L., Gordon, R.G., & Woodworth, D. Pacific plate apparent polar wander, hot spot fixity, and true polar wander during the formation of the Hawaiian Island and seamount chain from an analysis of the skewness of magnetic anomaly 20r (44 Ma). *Tectonics* **37**, 2094-2105 (2018).
10. Cox, A., & Gordon, R.G. Paleolatitudes determined from paleomagnetic data from vertical cores. *Rev. Geophys.* **22** 47-72 (1984).
11. Acton, G.D., & Gordon, R.G. A 65 Ma palaeomagnetic pole for the Pacific plate from the skewness of magnetic anomalies 27r-31. *Geophys. J. Inter.* **106**, 407-420 (1991).
12. Petronotis, K.E., Gordon, R.G., & Acton, G.D. A 57 Ma Pacific plate palaeomagnetic pole determined from a skewness analysis of crossings of marine magnetic anomaly 25r. *Geophys. J. Int.* **118**, 529-554 (1994).
13. Horner-Johnson, B.C. & Gordon, R.G. True polar wander since 32 Ma B.P.: A paleomagnetic investigation of the skewness of magnetic anomaly 12r on the Pacific plate. *J. Geophys. Res.* **15**, B09101 (2010).
14. Koivisto, E.A., Gordon, R.G., Dyment, J., & Arkani-Hamed, J. The spreading-rate dependence of anomalous skewness of Pacific plate magnetic anomaly 32: Revisited. *Lithosphere* **3**, 371-378 (2011).
15. Tarduno, J. A., Bunge, H.-P., Sleep, N. & Hansen, U. The Bent Hawaiian-Emperor hotspot track: Inheriting the mantle wind. *Science* **324**, 50-53 (2009).
16. Tarduno, J.A. On the motion of Hawaii and other mantle plumes. *Chemical Geol.* **241**, 234-247 (2007).
17. Cande, S.C., Raymond, C.A., Stock, J. & Haxby, W.F. Geophysics of the Pitman fracture zone and Pacific-Antarctic plate motions during the Cenozoic. *Science* **270**, 947-953 (1995).
18. Steinberger, B., Sutherland, R. & O'Connell, R.J. Prediction of Emperor-Hawaii seamount locations from a revised model of global plate motion and mantle flow. *Nature* **430**, 167-173 (2004).
19. Whittaker, J.M., Williams, S.E. & Müller, R.D., Revised tectonic evolution of the Eastern Indian Ocean. *Geochem. Geophys. Geosyst.* **14**, 1891-1909 (2013).
20. Müller, R.D *et al.* Ocean basin evolution and global-scale plate reorganization events since Pangea breakup. *Annual Rev. Earth Planet. Sci.* **44**, 107-138 (2016).

21. Doubrovine, P. V. & Tarduno, J.A. A revised kinematic model for the relative motion between Pacific oceanic plates and North America since the Late Cretaceous. *J. Geophys. Res.* **113**, B12101 (2008).
22. Doubrovine, P. V. & Tarduno, J.A. Linking the Late Cretaceous to Paleogene Pacific plate and the Atlantic bordering continents using plate circuits and paleomagnetic data. *J. Geophys. Res.* **113**, B07104 (2008).
23. Konrad, K., *et al.* On the relative motions of long-lived Pacific mantle plumes. *Nature Commun.* **9**, 854 (2018).
